# High-order finite element methods for cardiac monodomain simulations

**DOI:** 10.3389/fphys.2015.00217

**Published:** 2015-08-05

**Authors:** Kevin P. Vincent, Matthew J. Gonzales, Andrew K. Gillette, Christopher T. Villongco, Simone Pezzuto, Jeffrey H. Omens, Michael J. Holst, Andrew D. McCulloch

**Affiliations:** ^1^Department of Bioengineering, University of California San DiegoLa Jolla, CA, USA; ^2^Department of Mathematics, University of ArizonaTucson, AZ, USA; ^3^Dipartimento di Matematica, Politecnico di MilanoMilano, Italy; ^4^Center for Computational Medicine in Cardiology, Institute of Computational Science, Università della Svizzera italianaLugano, Switzerland; ^5^Department of Medicine, University of California San DiegoLa Jolla, CA, USA; ^6^Department of Mathematics, University of California San DiegoLa Jolla, CA, USA

**Keywords:** finite element analysis, monodomain model, serendipity methods, cell Thiele modulus, cardiac activation pattern

## Abstract

Computational modeling of tissue-scale cardiac electrophysiology requires numerically converged solutions to avoid spurious artifacts. The steep gradients inherent to cardiac action potential propagation necessitate fine spatial scales and therefore a substantial computational burden. The use of high-order interpolation methods has previously been proposed for these simulations due to their theoretical convergence advantage. In this study, we compare the convergence behavior of linear Lagrange, cubic Hermite, and the newly proposed cubic Hermite-style serendipity interpolation methods for finite element simulations of the cardiac monodomain equation. The high-order methods reach converged solutions with fewer degrees of freedom and longer element edge lengths than traditional linear elements. Additionally, we propose a dimensionless number, the cell Thiele modulus, as a more useful metric for determining solution convergence than element size alone. Finally, we use the cell Thiele modulus to examine convergence criteria for obtaining clinically useful activation patterns for applications such as patient-specific modeling where the total activation time is known *a priori*.

## Introduction

Detailed tissue-scale cardiac electrophysiology simulations are commonly used to investigate the dynamics and mechanisms of arrhythmias (Sato et al., 2009; Moreno et al., 2011; Trayanova, 2014) and drive electromechanical simulations (Campbell et al., 2009; Aguado-Sierra et al., 2011; Niederer et al., 2011; Trayanova, 2011). Beyond the inherent physiological complexity, these models pose numerous numerical and computational challenges. For example, insufficient spatial resolution can lead to spurious behavior including artifactual breakup of reentrant waves (Krishnamoorthi et al., 2013), a lack of wavebreak (Bueno-Orovio et al., 2008), or pinning of reentrant rotors to the computational grid (Fenton et al., 2002). Thus, independent verification of electrophysiology solvers is essential, particularly as models move closer to clinical applications.

Niederer et al. (2011a) provided a benchmark model for cardiac monodomain electrophysiology solvers that included anisotropic propagation and realistic human ventricular action potential kinetics. Previous studies had suggested that discretization to a spatial scale of 0.25 mm was sufficient for converged action potential propagation (Cherry and Fenton, 2004; Xie et al., 2004; Clayton and Panfilov, 2008). However, in the community benchmark exercise, none of the solution schemes were converged to within 5% of the consensus total activation time with element edge lengths of 0.2 mm, and many methods, including all solvers using hexahedral element meshes, deviated from the consensus solution by more than 10% at a spatial discretization of 0.1 mm (Niederer et al., 2011a). This important report suggested that reliable electrophysiology simulations may require more highly refined spatial discretizations than previously thought.

The benchmark exercise also provided insight into the convergence behavior of different numerical schemes. A subsequent investigation identified mass lumping as the primary culprit for underestimated propagation speeds with larger spatial steps or element sizes in several solvers (Pathmanathan et al., 2012). Another study identified combinations of lumping schemes and quadrature rules that provided somewhat more accurately converged results with increased computational efficiency (Krishnamoorthi et al., 2013). High-order methods have been proposed to take advantage of their potential convergence benefits (Arthurs et al., 2012, 2013; Cantwell et al., 2014). However, these reports did not evaluate cubic Hermite finite element schemes, which have long been popular for modeling cardiac geometry (Nielsen et al., 1991), electrophysiology (Rogers and McCulloch, 1994) and biomechanics (Costa et al., 1996).

Here we use the 2011 benchmark problem and a biventricular model to examine the convergence of a Galerkin finite element formulation of the monodomain problem using three types of elements on hexahedra: tri-linear Lagrange, tri-cubic Hermite, and new cubic order Hermite-style serendipity elements. The serendipity element has significantly fewer basis functions than the tri-cubic element but still conforms to recent mathematical theory regarding cubic order convergence (Arnold and Awanou, 2011, 2014; Gillette, 2014).

The convergence of numerical electrophysiology solutions depends not only on element size but also on other properties including the conductivity (Pollard et al., 1993; Rogers and McCulloch, 1994) and ion channel kinetics (Bernus et al., 2002). These intuitive relationships are commonly used to tune conduction velocity (Costa et al., 2013) and have been leveraged to reduce computational expense (Bernus et al., 2002). However, the relationship between these parameters and convergence is not commonly reported. Therefore, we sought a dimensionless parameter that combines these properties into a more appropriate determinate of convergence than line length alone. We found that a version of the Thiele modulus, well known in transport theory as the ratio between the characteristic rates of reaction and diffusion (Thiele, 1939; Hill and Root, 2014), is a more complete metric to describe discretization error and solution stability in monodomain simulations. Finally, we used this dimensionless number to better define the convergence criteria and compare the relative performance of each basis type for a modeling application where the activation pattern is required and strict numerical convergence is not essential.

## Methods and models

### Governing equation—the monodomain equation

Electrical impulse propagation in the heart is commonly modeled using either the bidomain equation (Tung, 1978) or the monodomain equation (Keener and Sneyd, 1998). Here, as in Niederer et al. (2011a), we consider the monodomain equation. This reaction-diffusion type parabolic partial differential equation (PDE) is derived from the cable equation and states the conservation of charge. The “reaction” portion of the monodomain equation is a system of ordinary differential equations (ODEs) that represent the flux of ions across the myocyte membrane, and the “diffusion” portion of the monodomain equation is a PDE that represents the spread of current through gap junctions and across cardiac tissue. The monodomain equation is as follows:

(1) $$\begin{array}{l} \chi \left(C_{m}\frac{\partial u}{\partial t}+I_{ionic}\left(u\right)\right)=\nabla \cdot \sigma \nabla u \end{array}$$

where *u* is the transmembrane potential, σ is the conductivity tensor, *I*_*ionic*_ is the current due to the flow of ions through channels in the cell membrane, *C*_*m*_ is the specific capacitance of the cell membrane, and χ is the surface area to volume ratio. *I*_*ionic*_ is described by a system of ODEs where the current is a function of the voltage *u*, channel gating states, and other state variables.

### Basis functions

A Galerking finite element method was used for spatial discretization of the monodomain equation. The convergence of this numerical method with three types of finite element interpolation functions were evaluated: C_0_ continuous linear Lagrange interpolation in trilinear hexahedral elements with 8 nodes and 8 degrees of freedom; C_1_ continuous cubic Hermite interpolation in tricubic hexahedral elements with 8 nodes and 64 degrees of freedom; and newly derived C_0_ continuous cubic Hermite-style “serendipity” interpolation in tricubic Hermite elements that preserve agreement of the partial derivatives along mesh edges with 8 nodes and 32 degrees of freedom.

Serendipity spaces allow for a reduction in the number of degrees of freedom in a finite element problem while retaining the maximum order of convergence under appropriate regularity of the solution. Arnold et al. recently demonstrated that only *superlinear monomials* up to and including the degree *p* are sufficient conditions for *p*-order convergence (Arnold and Awanou, 2011, 2014). The superlinear degree of a monomial is its total degree, but ignoring any variables that appear with linear order. More recently, cubic Hermite-style serendipity basis functions were derived (Gillette, 2014), which are employed in the current work. The two-dimensional and three-dimensional cubic Hermite-style serendipity basis functions are provided in Supplementary Material. For the quadrilateral element, the number of degrees of freedom is reduced from 16 for bicubic Hermite interpolation to 12 for the bicubic Hermite-style serendipity interpolation; for the hexahedral element, the number is reduced from 64 to 32. For brevity, we will hereafter refer to cubic Hermite-style serendipity basis functions as “serendipity Hermite.”

### Cell thiele modulus

For reaction-diffusion problems, the Thiele modulus, used in chemical engineering, describes the ratio of the rate of reaction to the rate of diffusion (Thiele, 1939; Hill and Root, 2014). Analogous to the local or cell Péclet number for advection diffusion problems (Brooks and Hughes, 1982; Pullan et al., 2002; Quarteroni, 2009), we define the dimensionless cell Thiele modulus (ϕ_*c*_) as the ratio of the discretization length to the characteristic length of the monodomain equation with the form:

(2) $$\begin{array}{l} \phi_{c}\equiv h\sqrt{\frac{k}{D}} \end{array}$$

where *k* is the reaction rate (normalized dV/dt_*max*_, defined as dV/dt_*max*_ of the single cell ionic model divided the action potential amplitude), *h* is the mean element edge length, and *D* is the diffusivity along an eigenaxes of the conductivity tensor. A derivation of the cell Thiele modulus from the non-dimensionalization of the monodomain equation is provided in Supplementary Material.

### Solution scheme

Operator splitting (Sundnes et al., 2005) was employed to separate the reaction (ODE) and diffusion (PDE) components of the monodomain equation and solve them in series. The system of ODEs representing the cell ionic model was first updated using a single-iteration backwards Euler solver (Lionetti, 2010) with a fixed sub-stepping time step of 0.001 ms. The ODEs were evaluated at each Gauss-Legendre quadrature point in the computational mesh, and these calculations were accelerated by solving them on an NVIDIA GPU. The voltages at the quadrature points were then projected onto the basis functions. Thereafter, the coefficients defining the updated nodal voltages and their spatial derivatives were determined by solving the global linear system. A backwards Euler scheme with a fixed time step of 0.01 ms was employed for the PDE, and the linear solver was a conjugate gradient method implemented in PyTrilinos (Sala et al., 2008) using incomplete LU factorization for the preconditioner. Finally, the updated voltage solution was evaluated at the quadrature points before the next time step. The solver was implemented in the publicly available *Continuity* 6^1^ software package, which previously used a collocation finite element method to solve the monodomain equation (Rogers and McCulloch, 1994).

### Meshes and simulations

Numerical experiments were performed on three test problems. All simulations used the 2006 ten Tusscher cell ionic model (ten Tusscher and Panfilov, 2006) as described in the benchmark study (Niederer et al., 2011a), except as noted.

The first test problem was the benchmark problem defined by Niederer et al. (2011a). The mesh comprised a 20.0 × 7.0 × 3.0 mm cuboid of simulated cardiac tissue. The conductivity was transversely anisotropic with a conductivity of 0.133 mS/mm in the fiber direction (parallel to the long axis) and 0.0176 mS/mm along the other axes. A stimulus (2 ms at 50,000 μAcm^−3^) was applied to a 1.5 mm cube in one corner of the mesh. The error in the total activation time was computed from the midpoint of the consensus solution presented by Niederer et al. (42.75 ms).

The second test problem was a simplified version of the 2011 benchmark that produced a planar propagating wavefront. A uniform stimulus (2 ms at 50,000 μAcm^−3^) was applied to one end of the mesh, and average conduction velocity was measured using the activation times at 20 and 80% along the long axis of the mesh to avoid boundary effects. A series of nested cuboid meshes of the same aspect ratio was used to compare solutions across a large range of element edge lengths (0.05–8.0 mm) and nearly five orders of magnitude in degrees of freedom. This range was not possible by simple refinement of a single mesh. The conduction velocity measured using 0.05-mm cubic Hermite solution was used as the reference solution (0.62 m/s).

Finally, simulations were also performed in a three-dimensional anatomic model of the human ventricles using a previously published human biventricular geometry generated using clinical computed tomography data from a patient with left bundle branch block and dyssynchronous heart failure (Villongco et al., 2014). The transversely isotropic conductivity was 0.0346 mS/mm in the fiber direction and 0.00494 mS/mm in the cross-fiber and sheet directions. A stimulus (2 ms at 50,000 μAcm^−3^) was applied to a small region on the endocardium of the right ventricular free wall to simulate endocardial pacing.

## Results

**Figure 1:**
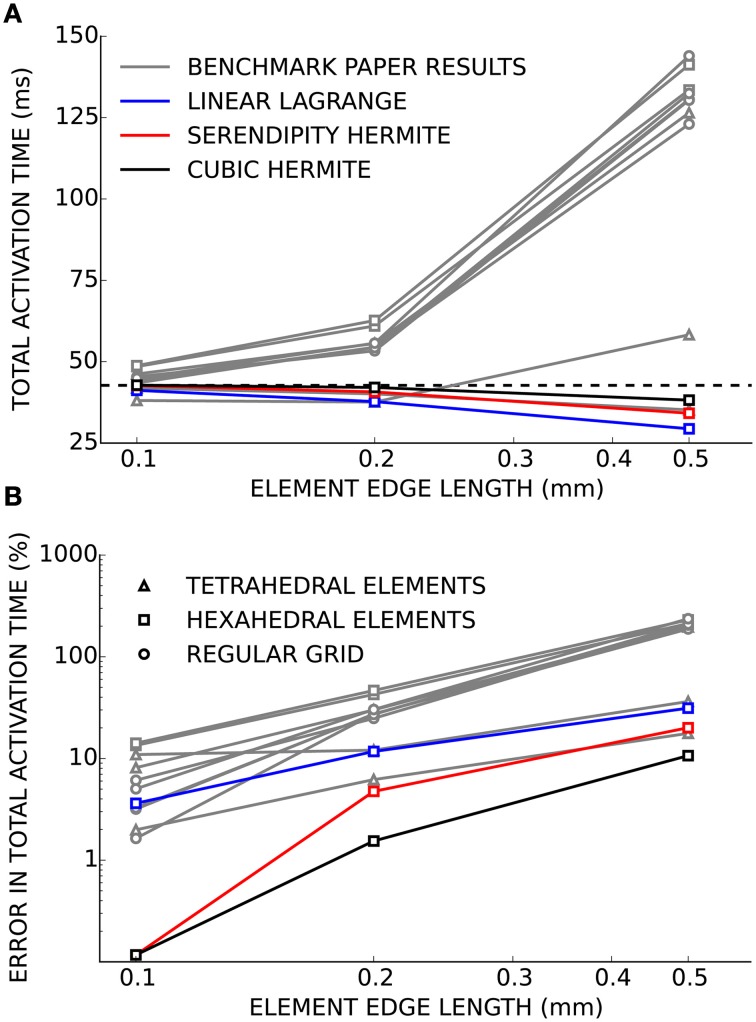
**(A)** Total activation time for simulations using cubic Hermite (black), cubic Hermite-style serendipity (red), and linear Lagrange (blue) elements all converge to the consensus total activation time (broken black line) for the cardiac electrophysiology benchmark problem. Gray lines represent solutions from the other solvers presented in Niederer et al. (2011a). Hexahedral (box), tetrahedral (triangle), or regular grid (circle) meshes are indicated by the marker style. In **(B)** the results are presented as the percent error in the total activation time.

Verification of our electrophysiology solver was performed using the benchmark problem. As the mesh was refined and the element edge length decreased, the solutions computed using linear Lagrange, serendipity Hermite, and cubic Hermite basis functions all converged to the consensus solution presented in the benchmark paper (Figure 1). At an element edge length of 0.2 mm, the error in the total activation times were 11.5, 4.7, and 1.5% using linear Lagrange, serendipity Hermite, and cubic Hermite elements, respectively. With a 0.1 mm element edge length, the linear Lagrange solver had an error in total activation time of 1.1% and both the serendipity Hermite and cubic Hermite solutions had errors of 0.1% and were within the uncertainty of the consensus solution. All three schemes converged from propagation speeds that were too fast.

**Figure 2:**
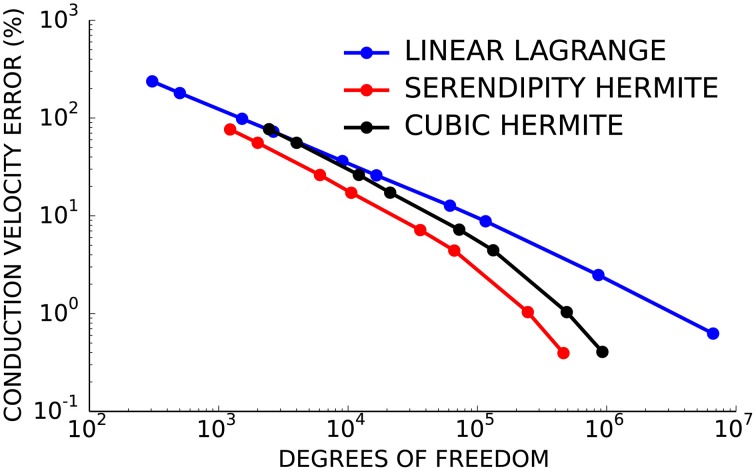
**Cubic Hermite-style serendipity elements (red) converge better per degree of freedom than conventional cubic Hermite (black) or linear Lagrange (blue) elements**.

Next, the convergence behavior of the three basis types was examined in more detail using the second test problem with planar wavefront propagation. Here, the serendipity Hermite solutions converged with fewer total degrees of freedom than the cubic and linear solutions (Figure 2). Solutions using the same number of cubic Hermite and serendipity Hermite elements were almost identical for this test problem.

**Figure 3:**
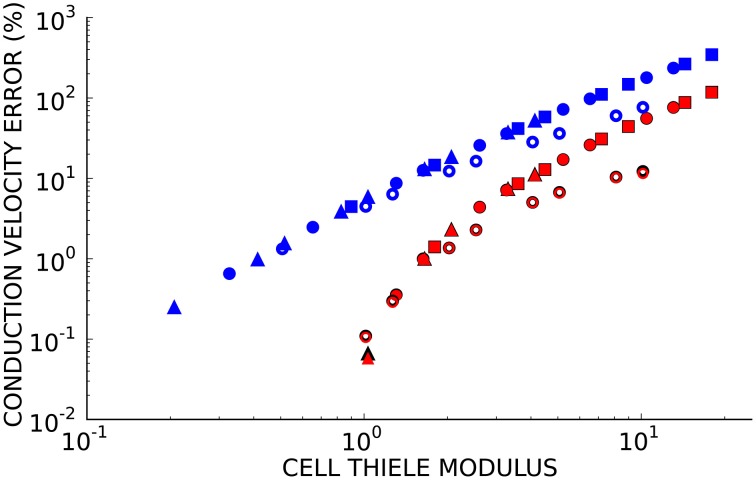
**The relationship between cell Thiele modulus and conduction velocity error is nearly constant for each basis function type**. Symbol color indicates interpolation type:cubic Hermite (black), cubic Hermite serendipity (red), and linear Lagrange (blue). Filled symbols represent solutions using the ten Tusscher (ten Tusscher and Panfilov, 2006) cellular ionic model and open symbols represent solutions using the Beeler-Reuter cellular ionic model (Beeler and Reuter, 1977). Diffusivity is represent by symbol shape: *D* = 0.0126 mm^2^/ms (boxes), *D* = 0.0953 mm^2^/ms (circles), *D* = 0.953 mm^2^/ms (triangles). Element lengths ranged from 0.05 to 4.0 mm.

Since numerical solutions of the monodomain model depend on membrane kinetics and monodomain conductivity as well as spatial discretization, we compared solutions to the second test problem using eight element sizes, three diffusivities (*D* = 0.0126, 0.0953, and 0.953 mm^2^/ms), two ionic models with different membrane kinetics (the ten Tusscher and Panfilov, 2006 human ventricular myocyte model with a normalized dV/dt_*max*_ of 2.47 ms^−1^ and the Beeler and Reuter (1977) ventricular myocyte model with a normalized dV/dt_*max*_ of 1.38 ms^−1^). Figure 3 demonstrates that the convergence error for these simulations is an almost unique function of cell Thiele modulus for each element type. A cell Thiele Modulus of 1.0 resulted in an error in the conduction velocity of approximately 0.1% with Hermite basis functions compared with 4% with linear Lagrange basis functions.

**Figure 4:**
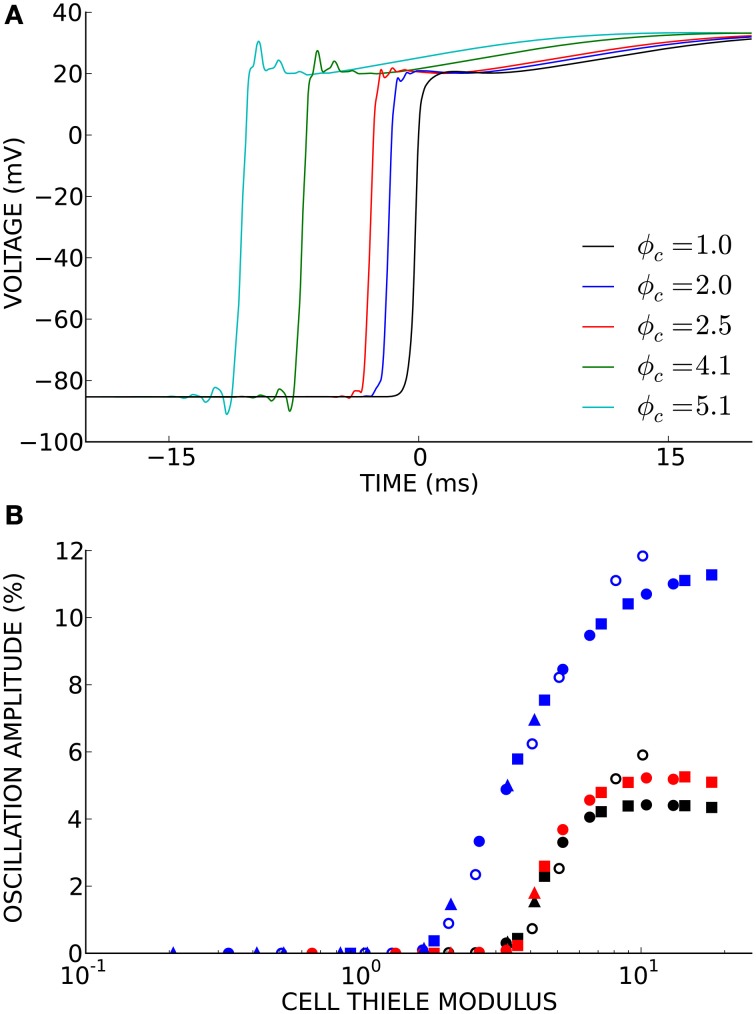
**(A)** Non-physiological oscillations were seen in unconverged solutions. Here *t* = 0 ms is set to the activation time of the fully converged (ϕ_*c*_ = 1.0) solution. **(B)** The amplitude of these oscillations is a function of the cell Thiele modulus. Symbol color indicates element type: cubic Hermite (black), cubic Hermite-style serendipity (red), and linear Lagrange (blue). Filled symbols represent solutions using the ten Tusscher (ten Tusscher and Panfilov, 2006) cellular ionic model and open symbols represent solutions using the Beeler-Reuter cellular ionic model (Beeler and Reuter, 1977). Diffusivity is represent by symbol shape: *D* = 0.0126 mm^2^/ms (boxes), *D* = 0.0953 mm^2^/ms (circles), *D* = 0.953 mm^2^/ms (triangles). Element lengths ranged from 0.05 to 4.0 mm.

Non-physiological oscillations were observed in unconverged solutions (Figure 4A). The amplitude of these oscillations, measured as the maximum negative deviation from resting membrane potential before the action potential upstroke, was a function of the cell Thiele modulus (Figure 4B). However, this relationship was much more non-linear than the relationship between conduction velocity error and cell Thiele modulus. The oscillation amplitude decreased sharply to zero around ϕ_*c*_ = 4.0 for the cubic Hermite and serendipity Hermite basis functions and were completely eliminated at ϕ_*c*_ ≲ 1.0.

**Figure 5:**
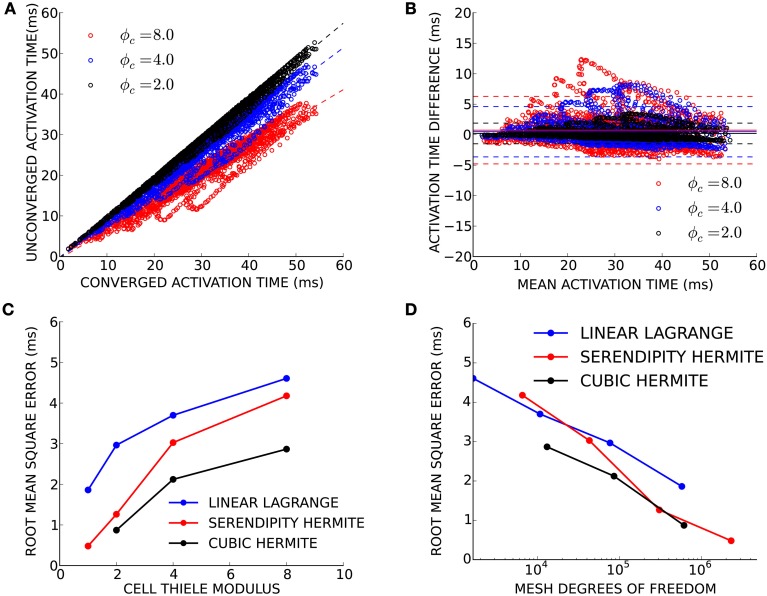
**(A)** Activation times from three unconverged electrophysiology simulations using cubic Hermite elements (black, ϕ_*c*_ = 2.0; blue, ϕ_*c*_ = 4.0; and red, ϕ_*c*_ = 8.0) are plotted against activation times at the same location on the mesh of a converged (ϕ_*c*_ = 1.0) reference solution. Regression lines are shown for each unconverged solution as a broken line. The activation times in the unconverged location were scaled so that regression line had a slope of 1.0. **(B)** These scaled activation times are compared with the converged activation times using a Bland-Altman plot where solid lines represent the mean difference between the two solutions and the broken lines are ± two standard deviations in the residuals from the regression line. **(C)** The root mean squared (RMS) error between the unconverged and fully converged activation patterns decreases with the cell Thiele modulus of the simulation. **(D)** The high-order methods have a smaller RMS error compared to the linear method on a degree of freedom basis particularly as the size of the problem increases.

Finally, we sought to determine convergence criteria for electrophysiology solutions when only activation patterns rather than absolute conduction times are needed. This need arises commonly in patient-specific modeling, where the total activation time is known (e.g., from a measured QRS duration or electrocardiological mapping), but the conductivity is unknown. Electrical propagation in a patient-derived human biventricular mesh was simulated at four levels of spatial refinement resulting in simulations with an average cell Thiele modulus in the primary direction of propagation of 1.0, 2.0, 4.0, and 8.0 for the four mesh refinements. For this exercise, the simulation using cubic Hermite elements with ϕ_*c*_ = 1.0 was considered fully converged. Activation times in the three less-converged simulations were compared to the converged activation times at node locations from the coarsest mesh (Figure 5A). Since propagation of the high cell Thiele modulus simulations was too fast, the total activation time for those simulations was scaled to give a regression line with a slope of 1.0. The Bland-Altman plot in Figure 5B comparing the scaled activation times and the fully converged activation times identifies a pattern of outliers (>2 standard deviations) that were too fast compared with the fully converged solution. These outliers were located in the basal right ventricular free wall. The root-mean-squared (RMS) error between fully converged activation pattern and the scaled unconverged activation patterns decreases as the cell Thiele modulus decreases toward 1.0 for all three interpolation methods (Figure 5C). The RMS error was less than 5 ms for all unconverged simulations and twice the RMS error was less than 5 ms for the ϕ_*c*_ = 2.0 and ϕ_*c*_ = 4.0 simulations with cubic Hermite elements, the ϕ_*c*_ = 2.0 and ϕ_*c*_ = 1.0 simulations with serendipity Hermite elements, and the ϕ_*c*_ = 1.0 simulation with the linear Lagrange elements. This error is comparable to the lowest uncertainly in clinically measured activation times (Gold et al., 2011; Villongco et al., 2014). Figure 5D demonstrates that the high-order methods also have smaller RMS error than the linear elements on a degree of freedom basis.

## Discussion

The above results are in agreement with previous studies demonstrating higher convergence rates with cubic Hermite and other high-order basis functions (Rogers et al., 1997; Arthurs et al., 2012; Cantwell et al., 2014). The application of cubic Hermite-style serendipity basis functions and the cell Thiele modulus to the cardiac electrophysiology problem are, to our knowledge, novel contributions.

The solutions to the cardiac electrophysiology benchmark problem converged to the consensus total activation time and compare favorably to the other solvers reported by Niederer et al. (2011a). The behavior of the linear Lagrange solution suggests the convergence of hexahedral element meshes is not limited by their element choice. The slow conduction velocities exhibited at larger spatial scales by all of the solvers using hexahedral element meshes in the Niederer et al. paper were likely due to their choice of mass lumping as suggested by Pathmanathan et al. (2012), and not the type of element. Solutions obtained using cubic Hermite and serendipity Hermite basis functions had substantially less error in total activation time at the same spatial discretization compared with our linear Lagrange solution and with solutions from the solvers tested by Niederer et al. (2011a). At 0.5 mm element size, Hermite solutions were as close to the consensus solution as most of the other solvers achieved at 0.1 mm discretization. This is unsurprising due to their additional degrees of freedom, but it also suggests that the 0.1 mm element edge length recommended by the benchmark exercise may be an overly stringent criterion. Results from Krishnamoorthi et al. (2013) suggest that our choice to integrate the cell ionic model at the Gauss-Legendre points (as opposed to at the nodes) was responsible for fast conduction in unconverged solutions in contrast to most of the solvers used in the benchmarking exercise.

A direct comparison of the three element types demonstrated that solutions using serendipity Hermite basis function have a convergence advantage per degree of freedom compared with linear Lagrange and cubic Hermite elements. These new cubic Hermite-style serendipity methods, applied to the cardiac electrophysiology problem for the first time here, balance the convergence benefits of high-order interpolation with lower computational demands.

Discussions of convergence criteria in cardiac electrophysiology simulations have focused on the element edge lengths (Rogers and McCulloch, 1994; Clayton and Panfilov, 2008; Niederer et al., 2011a) despite a well-known dependence of the solutions on the conductivity (Pollard et al., 1993; Rogers et al., 1997; Costa et al., 2013) and the stiffness of the cellular ODE model (Bernus et al., 2002). We have introduced a new dimensionless parameter analogous to the Thiele modulus from mass transport theory that can be used to specify convergence criteria. The cell Thiele modulus compares the discretization length to the characteristic length of the monodomain equation. The characteristic length of the monodomain equation is the square root of reaction rate over the diffusivity (see Supplementary Material). For simplified ionic currents where the reaction rate is clearly defined (i.e., the bistable equation where the ionic model is a cubic function), the characteristic length is the width of the propagating wavefront. Rescaling the non-linear ionic current component monodomain equation is more complex with a realistic cellular model, and we have defined the rate of reaction as the normalized *dV/dt*_*max*_ of the ionic model with units of ms^−1^. Alternatively, the reaction rate could be selected as the maximum time-varying conductance of the fast sodium channel (*g*_*na*_*(u)*; units of pS/nF or equivalently ms^−1^). This would shift the cell Thiele modulus results but not change the relationship as *dV/dt*_*max*_ and *g*_*na*_*(u)*_*max*_ are closely related. The components of this metric are often reported in papers, but in isolation they provide an incomplete description of the convergence. The cell Thiele modulus is more complete measure of the numerical convergence and stability.

We demonstrated that the cell Thiele modulus is proportional to the wave speed error and can be used to determine the presence of non-physiological oscillations in poorly converged solutions. The sharp decrease in oscillation amplitude with decreasing cell Thiele modulus and the corresponding low wave speed errors suggest that a lack of oscillations is a good criterion for convergence. The oscillations are a manifestation of the Gibbs phenomenon (Gottlieb and Shu, 1997). Methods to eliminate these oscillations include mass lumping and modified ODE integration schemes (Torabi Ziaratgahi et al., 2014). It has been suggested that these methods may not improve the underlying numerical stability of the solutions (Gresho and Lee, 1981). The cell Thiele modulus threshold where oscillations disappear is dependent on the order of the solution scheme. Our results suggest that a cell Thiele modulus of 3.0 is a reliable convergence and stability criterion when using the Hermite and serendipity Hermite elements and a cell Thiele modulus of 1.0 is sufficient for linear Lagrange elements. Additionally, by quantifying the discretization error and solution stability (i.e., the presence or absence of oscillations), the cell Thiele modulus could provide reasonable bounds during parameterization or optimization and could help guide adaptive schemes especially in the presence or anisotropic conduction or heterogeneous conductivities.

The small spatial discretization often cited for fully converged solutions can become computationally limiting particularly for applications such as patient-specific modeling (Villongco et al., 2014), that may necessitate parameter optimizations requiring hundreds or thousands of simulations. In these problems the total activation time is known whereas the conductivity is typically unknown and the available data may be too limited to allow highly detailed representations of the conducting system, ionic currents or tissue microstructure. We found that fully converged electrophysiology solutions were not required to obtain activation patterns to within clinically practical tolerances when the total activation time was known. For test simulations in a human biventricular model, activation patterns with RMS errors less than 2.5 ms were obtained using a cell Thiele modulus as high as 4.0 with cubic Hermite elements. The greatest errors were found on the basal right ventricular free wall where activation was mainly due to crossfiber conduction.

The simulations run at a high cell Thiele modulus can provide a reliable activation pattern but cannot guarantee biophysical fidelity due to the presence of oscillations. The utility of such simulations is therefore limited to applications such as the one described above where only the activation pattern is required. Other computational approaches to determine activation patterns, such as eikonal methods (Pullan et al., 2002), could be considered for this application. However, the monodomain solutions may be preferred especially if they are part of a coarse grain parameter optimization for subsequent fine grain simulations. Finally, the specific solution scheme presented here may not be optimal for conduction velocity tuning schemes as increased discretization error speeds up propagation necessitating decreased conductivities, and decreasing the conductivity to account for fast conduction will further increase the cell Thiele modulus

### Limitations

The above results compare the numerical convergence for an electrophysiology solver using different basis functions. However, the rate of numerical convergence is not the only consideration when comparing solution schemes. High-order Hermite methods are necessarily more complex to implement, and the optimal selection of a preconditioner for the linear system arising from the use of high-order methods is a long-standing issue in numerical analysis due to the poor condition number (relative to linear methods). The generation of high quality cubic Hermite hexahedral meshes for cardiac geometries from medical imaging data is a challenging problem and the resulting workflows may be more complex than automated methods for linear tetrahedral meshes (Zhang et al., 2012; Gonzales et al., 2013). Simulation time is also an important consideration, particularly for clinical applications. However, solution times are dependent on hardware, code optimization and architecture. The accuracy and stability of a numerical scheme as presented here on a degree of freedom basis is independent of the performance of a specific solver. Finally, as discussed in Costa et al. (2013), uncertainties introduced by numerical error may be minimal relative to the uncertainties in the parameterization of the models from physiological data. However, numerical error is problematic in biophysically detailed studies, as instabilities (i.e., the oscillations observed in Figure 4) will alter the cellular dynamics.

## Conclusions

The current analysis demonstrates that very small spatial discretizations (0.1 mm) are not necessarily required for well converged cardiac electrophysiology simulations under a variety of conditions. High-order cubic Hermite and cubic Hermite-style serendipity elements reached converged solutions with fewer degrees of freedom and longer element edge lengths than traditional linear elements. We suggest that since convergence also depends on membrane kinetics and conductivity parameters, the cell Thiele modulus is a more useful metric for determining solution convergence than element size alone. Finally, we found that clinically useful activation patterns can be obtained from cardiac electrophysiology solutions that are not close to being fully converged in situations such as patient-specific modeling where the total activation time is known *a priori*.

### Conflict of interest statement

ADM is a co-founder and equity holder in Insilicomed, Inc., a licensee of UCSD intellectual property used in this research that had no involvement in the work reported here. This relationship has been disclosed to the UCSD Independent Review Committee and is overseen by a Conflict of Interest Management Subcommittee of the IRC. The authors declare that the research was conducted in the absence of any commercial or financial relationships that could be construed as a potential conflict of interest.

## Footnotes

^1^www.continuity.ucsd.edu
