# Supplementary material for: High-order finite element methods for cardiac monodomain simulations
Source: Front Physiol. 2015 Aug 5;6:217. doi: 10.3389/fphys.2015.00217 (PMC4525671; doi:10.3389/fphys.2015.00217)
Supplement: Supplementary file 1 [file DataSheet1.DOCX]

**Appendix I**

The three-dimensional cubic Hermite-style serendipity basis functions are provided below for parametric coordinates ***ξ***_1_, ***ξ***_2_, and ***ξ***_3_ each on the interval [0,1]

where the superscripted number of **Ψ** indicates the node number and the subscripted number of **Ψ** indicates the basis function corresponding to the value (1), directional derivative of ***ξ***_1_ (2), the directional derivative of ***ξ***_2_ (3), or the directional derivative of ***ξ***_3_ (4). It is notable that the basis functions corresponding to the values $\boldsymbol{\Psi}_{1}^{(i)}$ can no longer be split into a product of separate functions of ***ξ***_1_, ***ξ***_2_, and ***ξ***_3_, i.e., their tensor product nature is lost. A consequence of this is that C_1_ continuity cannot be guaranteed between neighboring finite elements in the general case.

Using the same notation, the two-dimensional cubic Hermite-style serendipity basis functions are

$$\Psi_{\text{ser,2D}}=\left[\begin{matrix} \Psi_{1}^{(1)} \\ \Psi_{2}^{(1)} \\ \Psi_{3}^{(1)} \\ \Psi_{1}^{(2)} \\ \Psi_{2}^{(2)} \\ \Psi_{3}^{(2)} \\ \Psi_{1}^{(3)} \\ \Psi_{2}^{(3)} \\ \Psi_{3}^{(3)} \\ \Psi_{1}^{(4)} \\ \Psi_{2}^{(4)} \\ \Psi_{3}^{(4)} \end{matrix}\right]=\left[\begin{matrix} \\ -(\xi_{1}-1) (\xi_{2}-1) (-1-\xi_{1}+2\xi_{1}^{2}-2\xi_{2}+2\xi_{2}^{2}) \\ -\xi_{1} (\xi_{1}-1)^{2}(\xi_{2}-1) \\ -\xi_{2} (\xi_{1}-1) (\xi_{2}-1)^{2} \\ \xi_{1} (\xi_{2}-1) (-3\xi_{1}+2\xi_{1}^{2}-\xi_{2}+2\xi_{2}^{2}) \\ -\xi_{1}^{2} (\xi_{1}-1) (\xi_{2}-1) \\ \xi_{1}\xi_{2} (\xi_{2}-1)^{2} \\ \xi_{2} (\xi_{1}-1) (-\xi_{1}+2\xi_{1}^{2}-3\xi_{2}+2\xi_{2}^{2}) \\ \xi_{1}\xi_{2} (\xi_{1}-1)^{2} \\ -\xi_{2}^{2} (\xi_{1}-1) (\xi_{2}-1) \\ -\xi_{1}\xi_{2} (\xi_{1}-1) (-\xi_{1}+2\xi_{1}^{2}-3\xi_{2}+2\xi_{2}^{2}) \\ \xi_{1}^{2}\xi_{2} (\xi_{1}-1) \\ \xi_{1}\xi_{2}^{2} (\xi_{2}-1) \end{matrix}\right]$$

**Appendix II**

Below is a derivation of the cell Thiele modulus by non-dimensionalization of the monodomain equation. We begin with the monodomain equation:

$$\chi\left( C_{m}\frac{\partial u}{\partial t}+I_{ionic}\left( u \right) \right)=\frac{\partial}{\partial x}\left( \boldsymbol{\sigma}\frac{\partial u}{\partial x} \right)$$

We then scale the monodomain equation by the characteristic time, τ_m_, and characteristic length, λ_m_:

$$t⟼\frac{t}{\tau_{m}}$$

$$\frac{\partial u}{\partial t}⟼\frac{1}{\tau_{m}}\frac{\partial u}{\partial t}$$

$$x⟼\frac{x}{\lambda_{m}}$$

$$\frac{\partial^{2}u}{{\partial x}^{2}}⟼\frac{1}{{\lambda_{m}}^{2}}\frac{\partial^{2}u}{{\partial x}^{2}}$$

Replacing the nonlinear ionic current term with a normalized current term, *f(u)*, and the maximum rate of reaction, *k*.

$$I_{ionic}\left( u \right)=kf\left( u \right)$$

and substituting back into the monodomain equation we obtain:

$$\frac{1}{k\tau_{m}}\frac{\partial u}{\partial t}+f\left( u \right)=\frac{1}{kC_{m}\chi{\lambda_{m}}^{2}}\boldsymbol{\sigma}\frac{\partial^{2}u}{{\partial x}^{2}}$$

The conductivity tensor can be replaced by the diffusivity in direction of wave propogation, D.

$$D=\frac{1}{C_{m}\chi}\boldsymbol{\sigma n\cdot n}$$

The characteristic time constant and characteristic length constant can then be selected to nondimensionalize the monodomain equation.

$$\tau_{m}=\frac{1}{k}$$

$$\lambda_{m}=\sqrt{\frac{D}{k}}$$

$$\frac{\partial u}{\partial t}+f\left( u \right)=\frac{\partial^{2}u}{{\partial x}^{2}}$$

Finally, we define the cell Thiele modulus as the ratio of the discretization length, *h*, to the characteristic length.

$$\phi_{c}\equiv\frac{h}{\lambda_{m}}=h\sqrt{\frac{k}{D}}$$
